# Disputing your roots: A multi-platform computational analysis of consumer reactions to genetic ancestry testing

**DOI:** 10.1007/s12687-026-00903-w

**Published:** 2026-05-29

**Authors:** Sara Behnamian

**Affiliations:** Section for GeoGenetics, Globe Institute, University of Copenhagen, Copenhagen, Denmark

**Keywords:** Direct-to-consumer genetic testing, Ancestry testing, Natural language processing, Sentiment analysis, Consumer genomics, Social media analysis

## Abstract

Direct-to-consumer (DTC) genetic ancestry testing has grown rapidly, yet computational analysis of consumer reactions remains limited. This study presents a cross-platform computational analysis of consumer reactions to ancestry testing across 58,133 posts from Reddit, YouTube, and Google Play. We developed a six-category reaction taxonomy (acceptance, excitement, dispute, surprise, disappointment, identity crisis) and applied natural language processing methods including sentiment analysis, topic modeling, and predictive modeling. Results revealed that acceptance (9.5%) and excitement (9.4%) were most prevalent, followed by dispute (8.6%). Platform differences emerged: Reddit showed highest dispute rates (10.2%), while Google Play exhibited elevated excitement (29.6%). Dispute rates varied substantially by ancestry, with Turkish (23.5%), Greek (19.7%), and Scandinavian (18.5%) ancestries most frequently contested. Among posts containing both self-reported ethnicity and genetic results, concordance was 61.8%, quantifying the discrepancy between social and genetic definitions of ancestry. A logistic regression model predicting dispute expression achieved AUC = 0.79, identifying text length and negative sentiment as key predictors. These findings advance understanding of how consumers engage with genetic ancestry information online, with implications for DTC companies, genetic counselors, and researchers studying the social dimensions of consumer genomics.

## Introduction

Direct-to-consumer (DTC) genetic ancestry testing has experienced remarkable growth over the past decade, with more than 26 million consumers taking an at-home ancestry test by early 2019 (Regalado 2019). Companies such as 23andMe, AncestryDNA, and MyHeritage offer affordable autosomal DNA analysis that provides users with estimates of their biogeographical ancestry composition, often expressed as percentages attributed to various geographic regions or ethnic populations (Phillips 2016). These services have democratized access to genetic information that was previously available only through clinical or research settings, enabling individuals to explore questions of heritage, identity, and family history without professional intermediation (Harris et al. 2013).

The proliferation of DTC ancestry testing has generated significant scholarly interest in how consumers interpret, react to, and incorporate genetic ancestry information into their self-understanding. Survey-based research has documented that ancestry results can elicit a range of emotional responses, from excitement and validation to surprise, confusion, and psychological distress (Rubanovich et al. 2021; Schuman et al. 2024). Approximately 46% of users report experiencing surprise upon receiving their results, while a smaller but significant proportion (21%) indicate that results reshaped their sense of identity (Rubanovich et al. 2021). These findings suggest that ancestry information can influence how individuals interpret or revise their understandings of identity.

### Prior research on consumer responses to ancestry testing

Research examining consumer responses to DTC genetic ancestry testing has employed diverse methodological approaches. Survey-based studies have provided valuable quantitative insights into the prevalence and correlates of various reactions. Roberts et al. (2017) conducted a longitudinal survey finding that consumers expressed equal interest in ancestry and health information and reported high satisfaction with their testing experience. Rubanovich et al. (2021) followed a large cohort, documenting that while surprise was common (46%), severe distress was rare (< 1%). Systematic reviews have synthesized findings across multiple studies: Stewart et al. (2018) found that only a minority (under one quarter) of users reported positive lifestyle changes following DTC testing, and overall levels of psychological distress were low, while Roberts and Ostergren (2013) concluded that neither the expected benefits nor the feared harms of DTC testing materialized at the population level.

Qualitative interview studies have illuminated the deeper psychological and social dimensions of ancestry testing experiences (Table 1). Roth and Ivemark (2018) conducted in-depth interviews revealing that responses to ancestry results varied systematically by racial identity: white respondents often embraced unexpected results as opportunities to adopt new ethnic identities, while nonwhite respondents tended to maintain existing identifications regardless of genetic findings. This work underscored the socially constructed nature of genetic ancestry interpretation. More recent qualitative research has focused specifically on non-paternity events (NPE)—discoveries that one’s presumed biological father is not genetically related—documenting profound negative impacts on mental health, identity coherence, and family relationships (Careau et al. 2025; Becker et al. 2024; Grethel et al. 2022, 2024).

**Table 1:** Qualitative interview studies on consumer responses to DTC genetic ancestry testing

Study	Method	Population	Key Findings
Roth and Ivemark (2018)	In-depth interviews	Diverse racial groups	White respondents adopt new identities; nonwhite maintain existing identities
Careau et al. (2025)	Semi-structured interviews	NPE individuals	Profound negative mental health impact; identity disruption; family rupture
Becker et al. (2024)	Thematic analysis	NPE individuals	Identity impact; grief and loss; strained parent-child relationships
Grethel et al. (2022)	Thematic analysis	NPE individuals	Identity transformation; shifts in race, ethnicity, religion, belonging
Grethel et al. (2024)	Thematic analysis	NPE individuals	Disclosure dilemmas; stigma; emotional challenges
Lang and Winkler (2021)	Mixed methods	Experts and users	Companies and users co-construct ancestry meanings

*NPE *Non-Paternity Event (discovery that presumed father is not biological father)

A notably smaller body of research has applied computational text analysis methods to examine consumer discussions of genetic ancestry testing at scale (Table 2). To date, only a small number of NLP studies have examined this domain. Two prominent examples are Yin et al. (2020) and Toussaint et al. (2022). Yin et al. (2020) analyzed posts from Reddit communities (r/23andme and r/AncestryDNA), applying Latent Dirichlet Allocation (LDA) topic modeling and Linguistic Inquiry and Word Count (LIWC) sentiment analysis. They identified “Ancestral Origin” and “Kinship/Feelings” as the most discussed topics, with kinship-related discussions exhibiting the widest emotional range. Toussaint et al. (2022) examined YouTube comments using structural topic modeling and Bing/NRC sentiment lexicons, finding six primary topics and generally positive to neutral attitudes.

**Table 2:** Computational NLP studies on consumer discussions of DTC genetic ancestry testing

Study	Platform	Topic Modeling	Sentiment Analysis	Key Findings
Yin et al. (2020)	Reddit	LDA	LIWC	Kinship topics had widest emotional range
Toussaint et al. (2022)	YouTube	STM	Bing/NRC	6 topics; positive-neutral attitudes

*LDA *Latent Dirichlet Allocation, *STM* Structural Topic Modeling, *LIWC* Linguistic Inquiry and Word Count

### Gaps in the literature

Despite growing interest in consumer responses to genetic ancestry testing, several important gaps remain in the computational literature. Both existing NLP studies examined single platforms in isolation—Reddit (Yin et al. 2020) or YouTube (Toussaint et al. 2022)—precluding systematic comparison of how platform affordances shape consumer discourse. Different platforms attract distinct user populations and encourage different forms of expression: Reddit’s pseudonymous discussion forums may facilitate candid disclosure of sensitive reactions, while YouTube comment sections attached to “DNA reveal” videos may emphasize performative displays of surprise or excitement, and app store reviews likely reflect selection bias toward users motivated to provide feedback.

Furthermore, neither study systematically classified the types of emotional and cognitive reactions consumers express. While sentiment analysis captures overall positive or negative valence, it does not distinguish between qualitatively different responses such as surprise, dispute, acceptance, disappointment, identity disruption, or excitement. These reaction types likely differ in meaning, yet prior computational studies primarily focused on topic modeling and overall sentiment analysis rather than distinguishing discrete reaction categories (Yin et al. 2020; Toussaint et al. 2022).

The relationship between specific ancestry findings and consumer reactions has been examined only in limited qualitative work and remains underexplored computationally. Qualitative research suggests that certain ancestry results—particularly those involving Native American heritage—may be more likely to elicit skepticism or dispute (Walajahi et al. 2019). However, no study has extracted reported ancestry percentages from user posts or quantified dispute rates across ancestry categories. Similarly, existing research has not examined the concordance between self-reported ethnicity and genetic ancestry results in naturalistic online discourse, despite genetic anthropologists noting discrepancies between genetic and social definitions of ancestry (Morning 2011).

Finally, prior computational work has been primarily descriptive, documenting patterns in consumer discourse without developing predictive models. Understanding which post characteristics predict dispute expression could advance theory regarding the psychological and linguistic correlates of ancestry skepticism.

### The present study

This study addresses these gaps through a comprehensive computational analysis of consumer reactions to DTC genetic ancestry testing across multiple online platforms. We collected 58,133 user-generated posts from Reddit, YouTube, and Google Play, applying natural language processing methods to extract ancestry percentages, classify emotional reactions, analyze sentiment, discover latent topics, and model predictors of dispute expression.

Our approach builds on prior computational research in several ways. First, we provide a cross-platform analysis focused on consumer reactions across Reddit, YouTube, and Google Play. Second, we develop a six-category reaction taxonomy (surprise, dispute, acceptance, disappointment, identity crisis, excitement) that moves beyond binary sentiment to capture qualitatively distinct response types. Third, we extract reported ancestry percentages and normalize them into 42 standardized categories, allowing analysis of which ancestries are most frequently contested. Fourth, we compare users’ self-reported ethnicities with their genetic results, finding 61.8% concordance. Fifth, we develop a logistic regression model predicting dispute expression (AUC = 0.79), identifying text length and negative sentiment as key predictors. These contributions address fundamental gaps in understanding how consumers discuss and react to genetic ancestry information online.

Our analysis addresses five research questions: (1) How prevalent are different reaction types in consumer discussions of ancestry testing, and how do these distributions vary across platforms? (2) Which specific ancestries are most frequently contested, and what patterns emerge in ancestry-specific dispute rates? (3) What is the relationship between reaction type and sentiment, and do different sentiment analysis methods yield convergent results? (4) To what extent do users’ self-reported ethnicities align with their genetic ancestry results? (5) Which post characteristics predict expression of dispute?

## Methods

### Data collection

We collected user-generated content discussing direct-to-consumer (DTC) genetic ancestry testing from three major online platforms: Reddit, YouTube, and Google Play.

#### Reddit

Posts were scraped from four ancestry-related subreddits: r/23andme, r/AncestryDNA, r/ancestry, and r/Genealogy. Data retrieval used the Arctic Shift API (https://arctic-shift.photon-reddit.com/) as the primary source, with Pullpush API (https://pullpush.io/) as fallback. For each subreddit, we collected up to 10,000 posts sorted by creation date in descending order. Data were retrieved in December 2025. Reddit posts were collected using the Arctic Shift API (with Pullpush as fallback). The most recent Reddit posts available through the API at the time of retrieval were dated May 17, 2025, resulting in a dataset spanning August 2020 through May 2025. YouTube comments and Google Play reviews were also retrieved in December 2025 and span December 2016 through December 2025 (YouTube) and September 2024 through December 2025 (Google Play).

#### YouTube

Comments were extracted from ancestry DNA reveal videos using yt-dlp (https://github.com/yt-dlp/yt-dlp). We searched for videos using six query terms targeting reaction content. For each query, up to 30 videos were retrieved, and up to 200 comments were extracted per video.

#### Google Play

User reviews were collected using google-play-scraper (https://github.com/JoMingyu/google-play-scraper) from three major DTC genetic testing applications: AncestryDNA, 23andMe, and MyHeritage. Up to 1,000 reviews per app were retrieved, sorted by newest first.

All data were standardized into a common schema containing: unique identifier, source platform, text content, author, timestamp, and engagement metrics.

### Feature extraction

#### Ancestry percentage extraction

We developed regular expression patterns to identify reported ancestry percentages from free-text posts. Three pattern variants captured common reporting formats: “[percentage]% [ethnicity],” “[ethnicity]: [percentage]%,” and “[ethnicity] [percentage]%.” Extracted ethnicities were normalized into 42 standardized categories spanning six geographic regions (Table 3). Complete lexicons are available at https://github.com/sarabehnamian/ancestry_nlp.

**Table 3:** Ethnicity normalization categories by region

Region	Categories (*n*)	Examples of Lexicon Terms
European	12	British (British, English, Welsh, Scottish), Scandinavian (Swedish, Norwegian, Danish), Jewish (Ashkenazi, Sephardic)
African	7	Nigerian (Yoruba, Igbo, Hausa), West African (Senegalese, Malian), African American (Black American)
Asian	9	Chinese (Han), Indian (South Asian, Punjabi, Bengali), Southeast Asian (Thai, Indonesian)
Americas	8	Native American (Indigenous, Cherokee, Navajo), Mexican (Mestizo), Caribbean (Jamaican, Haitian)
Middle Eastern	3	Middle Eastern (Arab, Levantine, Iraqi, Syrian), Iranian (Persian), Turkish (Anatolian)
Oceania	3	Polynesian (Hawaiian, Samoan, Tongan), Melanesian (Fijian, Papua), Australian Aboriginal

#### Reaction classification

User reactions were classified into six categories using keyword-based pattern matching (Table 4). Each post received binary flags indicating presence or absence of each reaction type. Reaction categories were not treated as mutually exclusive. Each post received independent binary indicators for the presence of each reaction type. Consequently, posts expressing multiple or evolving reactions (e.g., initial excitement followed by disappointment) were coded as positive for all applicable categories. This approach reflects the mixed and dynamic nature of online discourse rather than imposing single-label classification.

**Table 4:** Reaction classification keywords

Reaction	Keywords
Surprise	surprised, shocking, shocked, unexpected, never expected, no idea, mind blown, wow, crazy, wild, insane, unbelievable
Dispute	wrong, incorrect, inaccurate, mistake, error, fake, scam, don’t believe, doubt, skeptical, no way, impossible
Acceptance	makes sense, expected, confirmed, accurate, correct, exactly what, as expected, no surprises, knew it, matches
Disappointment	disappointed, disappointing, wished, hoped for, wanted to be, thought I was, boring, basic, generic, letdown
Identity crisis	identity crisis, who am I, don’t know who, existential, questioning, lied to me, not my real, NPE, adoption
Excitement	excited, amazing, awesome, love, cool, fascinating, interesting, incredible, wonderful, thrilled, happy

Representative keywords shown; complete lexicons available at https://github.com/sarabehnamian/ancestry_nlp. These reaction categories were developed as operational discourse labels based on recurring lexical patterns in user-generated text rather than derived from established psychometric instruments. Because the study analyzes large-scale naturalistic online discussions, validated self-report scales could not be directly applied. These labels therefore represent text-based indicators of reaction types in discourse and should not be interpreted as validated psychological constructs

#### Self-reported ethnicity

was extracted using six pattern types targeting identity statements: “I’m/I am [ethnicity],” “I identify as [ethnicity],” “my family are/were [ethnicity],” “born/raised in [location],” “my ethnicity/heritage is [ethnicity],” and “I’m [ethnicity] American/Canadian/British.”

### Sentiment analysis

#### Lexicon-based sentiment scoring

We applied two complementary sentiment analysis methods. VADER (Valence Aware Dictionary and sEntiment Reasoner; Hutto and Gilbert 2014) computed compound sentiment scores ranging from − 1 (most negative) to + 1 (most positive), along with separate positive, negative, and neutral component scores. TextBlob (Loria 2018) provided supplementary polarity (− 1 to + 1) and subjectivity (0 to 1) measures. Posts were categorized as positive (compound > 0.05), neutral (− 0.05 to 0.05), or negative (compound < − 0.05) following VADER’s recommended thresholds (Hutto and Gilbert 2014).

The two tools were applied as a form of methodological triangulation rather than to generate independent inferential claims. VADER is optimized for short, informal social media text and captures intensity and negation patterns effectively, whereas TextBlob provides both polarity and subjectivity estimates. Using both allowed us to assess the robustness of polarity patterns across lexicons and to examine whether subjectivity provided additional interpretive insight beyond valence alone. Polarity estimates from VADER and TextBlob were moderately correlated (*r* = 0.411, *p* < 0.001), supporting convergent validity while indicating that the tools capture related but not identical dimensions of sentiment (Fig. 5).

#### Topic modeling

Latent Dirichlet Allocation (LDA; Blei et al. 2003) was applied to discover latent discussion themes. Text preprocessing included URL removal, non-alphabetic character removal, and lowercasing. Document-term matrices were constructed using scikit-learn (Pedregosa et al. 2011) with maximum document frequency of 0.95, minimum document frequency of 10, English stopword removal, and vocabulary limited to 5,000 features. The number of topics (k = 8) was selected based on qualitative interpretability of the resulting themes during exploratory modeling. We inspected top-loading terms and topic assignments to ensure that the solution produced coherent and distinguishable discussion themes without excessive overlap. The model was fit with 15 iterations.

#### Self-reported versus genetic ancestry comparison

For posts containing both self-reported ethnicity and genetic ancestry results, we computed concordance using a binary match variable. A post was coded as concordant (match = 1) if at least one shared normalized ancestry category appeared in both the self-reported and genetic ancestry sets; otherwise, it was coded as discordant (match = 0). Both self-reported and genetic ancestries were stored as comma-separated normalized categories and compared using set intersection. When users reported multiple identities (e.g., “I’m Irish and Italian”), all self-reported categories were compared against all genetic categories, requiring at least one shared category for a match. Fractional ancestry percentages were not incorporated into the concordance calculation; only the presence of normalized ancestry categories was considered. Posts lacking either self-reported ethnicity or genetic ancestry results were excluded from this analysis.

### Statistical Analysis

#### Association tests

Chi-square tests of independence assessed associations between categorical variables: presence of ancestry results and surprise reactions, presence of ancestry results and dispute reactions, and source platform and reaction types. Effect sizes were quantified using Cramér’s V. Statistical significance was set at α = 0.05.

#### Platform comparison

One-way analysis of variance (ANOVA) compared mean VADER compound sentiment scores across source platforms (Reddit, YouTube, Google Play). Effect size was quantified using eta-squared (η²). Post-hoc pairwise comparisons were conducted using Tukey’s Honestly Significant Difference (HSD) test (Tukey 1949).

#### Reaction-sentiment comparison

Independent samples t-tests compared sentiment scores between posts with and without each reaction type. For each reaction category, posts were divided into two groups based on the corresponding binary indicator (1 = reaction present; 0 = reaction absent), and mean VADER compound sentiment scores were compared between these groups. Effect sizes were quantified using Cohen’s d (Cohen 1988). Non-parametric Mann-Whitney U tests (Mann and Whitney 1947) were conducted as sensitivity analyses. Kruskal-Wallis H tests (Kruskal and Wallis 1952) compared sentiment distributions across all reaction types simultaneously.

#### Correlation analysis

Pearson correlation coefficients quantified relationships between continuous sentiment measures (VADER compound, TextBlob polarity, TextBlob subjectivity).

#### Dispute rate by ancestry

For each normalized ancestry category with at least 20 mentions, we calculated the proportion of posts containing that ancestry that also expressed dispute.

#### Predictive modeling

Dispute was selected as the target variable because it represents an active evaluative response to ancestry results, and understanding its predictors has practical relevance for DTC companies and genetic counselors seeking to anticipate consumer skepticism. Logistic regression modeled the probability of dispute expression as a function of four predictor variables selected to capture complementary dimensions of post content: text length (structural extent of the post), VADER compound sentiment score (affective tone), number of ancestries mentioned (result complexity), and top ancestry percentage (magnitude of the primary ancestry assignment). These features were chosen because they are extractable at scale from unstructured text and span structural, affective, and content-based properties of posts. Features were standardized using z-score normalization prior to model fitting. Model performance was evaluated using 5-fold cross-validation with area under the receiver operating characteristic curve (AUC) as the primary metric. All statistical analyses were conducted using SciPy (Virtanen et al. 2020) and scikit-learn (Pedregosa et al. 2011).

### Validation of reaction classification

To assess the accuracy of the keyword-based reaction classifier, we conducted a manual validation study on a stratified random subsample. A total of 300 posts were sampled from the corpus using stratified random sampling, with approximately 35 posts flagged positive for each of the six reaction types, 60 posts with no detected reaction, and additional randomly selected posts to reach the target sample size. This oversampling strategy ensured sufficient positive cases per category to compute reliable precision and recall estimates. One author (S.B.) independently annotated each post for all six reaction categories using a binary coding scheme (1 = present, 0 = absent). Annotations were guided by predefined definitions for each reaction type (see Table 4) and were based on the full semantic meaning of the text rather than the presence of individual keywords. One post was excluded due to incomplete annotation, yielding 299 fully coded posts. Classifier performance was evaluated using precision, recall, F1 score, Cohen’s κ, and accuracy for each reaction category and across all categories (micro- and macro-averaged). Cohen’s κ was selected as the primary agreement measure because it adjusts for chance agreement between the keyword classifier and human judgment.

## Results

### Dataset characteristics

The final corpus comprised 58,133 user-generated records from three platforms: Reddit (*n* = 40,000; 68.8%), YouTube (*n* = 17,133; 29.5%), and Google Play (*n* = 1,000; 1.7%). Reddit data originated from four subreddits: r/23andme, r/AncestryDNA, r/ancestry, and r/Genealogy. YouTube comments were extracted from ancestry DNA reveal videos. Google Play reviews were collected from three DTC applications: AncestryDNA, 23andMe, and MyHeritage (Table 5).

**Table 5:** Dataset composition by source platform

Platform	Records	Percentage
Reddit	40,000	68.8%
YouTube	17,133	29.5%
Google Play	1,000	1.7%
Total	58,133	100%

### Ancestry percentage extraction

**Fig. 1:**
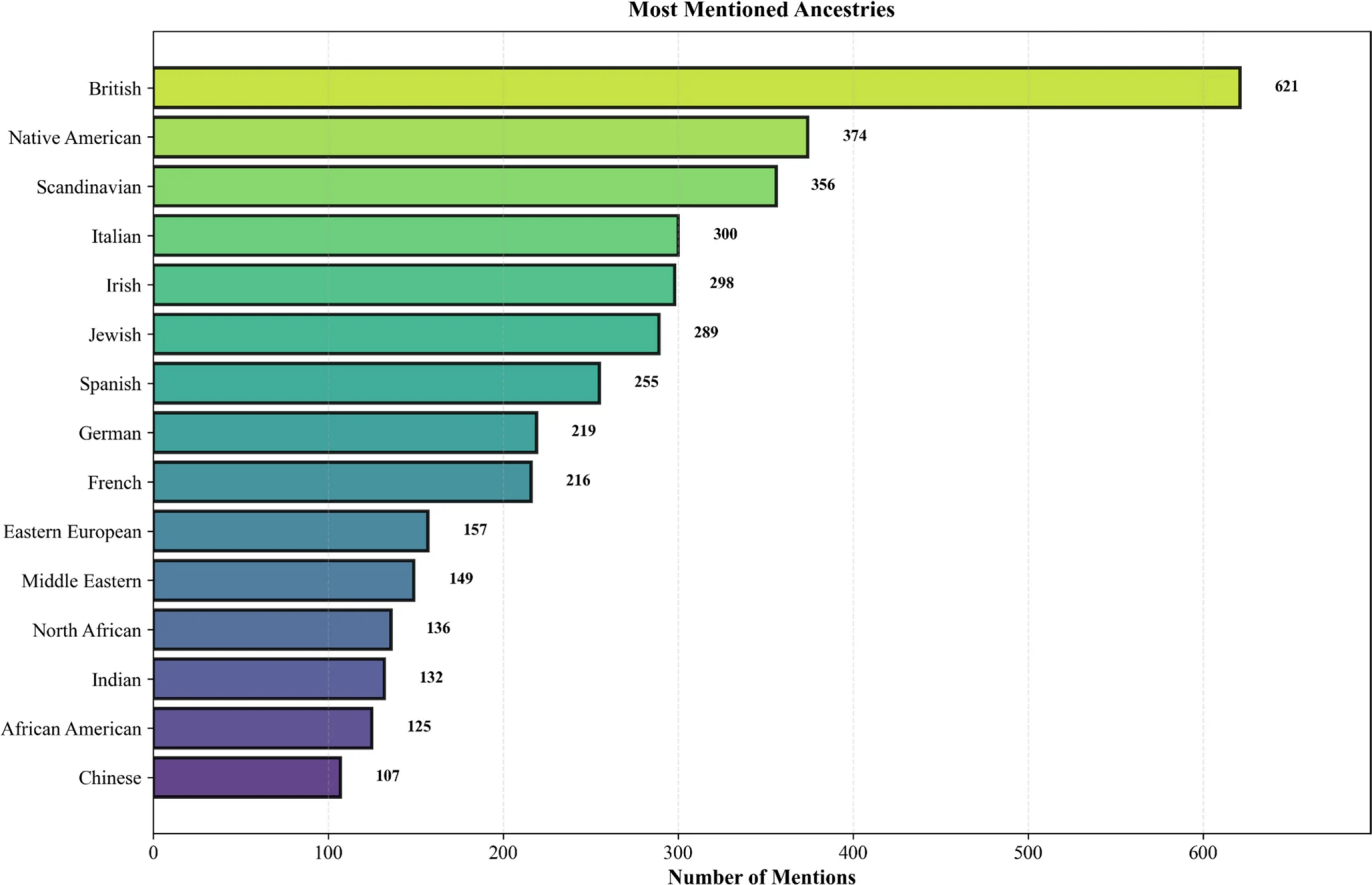
Most frequently mentioned ancestries across posts containing extractable ancestry percentages. European ancestries predominated, with British (*n* = 621) and Scandinavian (*n* = 356) among the most common. Native American ancestry ranked third overall (*n* = 374), followed by Jewish ancestry (*n* = 289). Bar heights represent raw mention counts across the corpus

Extractable ancestry percentages were identified in 4,379 posts (7.5% of corpus). Posts containing ancestry results reported a mean of 2.3 ancestry categories (SD = 1.8), with total percentages averaging 94.2% per post. European ancestries predominated: British (*n* = 621), Scandinavian (*n* = 356), Italian (*n* = 300), and Irish (*n* = 298). Native American ancestry ranked third overall (*n* = 374), followed by Jewish ancestry (*n* = 289) (Fig. 1).

### Self-reported ethnicity

Self-reported ethnicity was extracted from 978 posts (1.7%). The lower extraction rate relative to ancestry percentages indicates that users predominantly shared genetic test results rather than explicit self-identification statements.

### Reaction prevalence

Reaction classification identified emotional responses in 14,472 posts (24.9%). Acceptance was the most prevalent reaction (*n* = 5,546; 9.5%), followed by excitement (*n* = 5,453; 9.4%) and dispute (*n* = 4,985; 8.6%). Surprise occurred in 3,098 posts (5.3%), disappointment in 2,077 posts (3.6%), and identity crisis in 1,313 posts (2.3%). Posts exhibited a mean of 0.43 reaction types, indicating co-occurrence of multiple reactions (Table 6).

**Table 6:** Reaction prevalence across corpus

Reaction	*n*	Percentage
Acceptance	5,546	9.5%
Excitement	5,453	9.4%
Dispute	4,985	8.6%
Surprise	3,098	5.3%
Disappointment	2,077	3.6%
Identity crisis	1,313	2.3%

### Platform differences

**Fig. 2:**
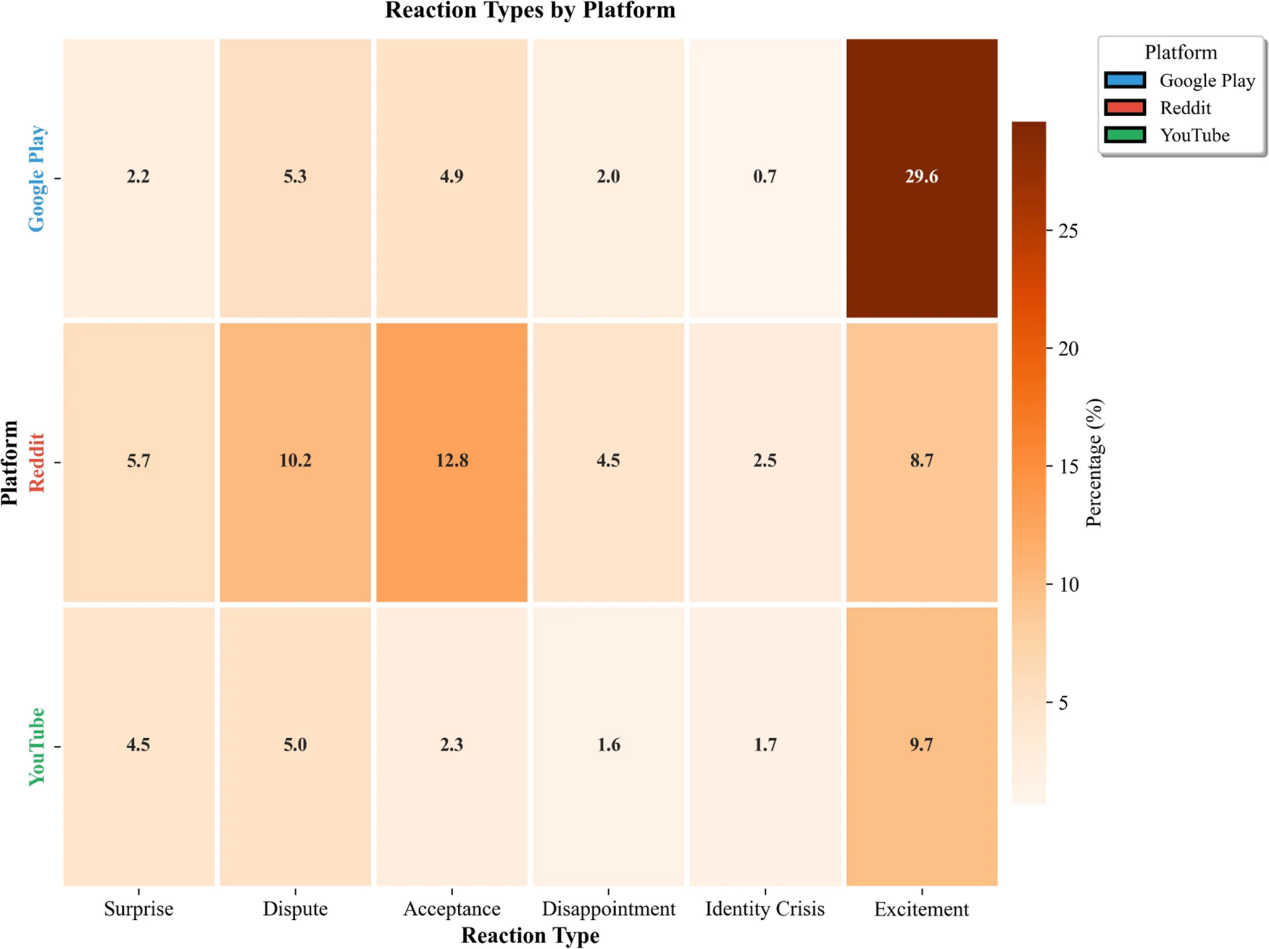
Reaction type prevalence by platform. Reddit exhibited the highest dispute (10.2%) and acceptance (12.8%) rates, reflecting the platform’s affordance for detailed discussion. Google Play showed elevated excitement (29.6%), consistent with selection bias toward satisfied users providing app reviews. YouTube comments demonstrated intermediate patterns across all reaction types

Reaction distributions differed across platforms (Fig. 2). Reddit exhibited higher dispute rates (10.2%) and acceptance rates (12.8%) compared to YouTube. YouTube comments demonstrated elevated excitement (9.7%). Google Play reviews showed the highest excitement rates (29.6%), consistent with selection bias toward satisfied users providing reviews.

### Company mentions

Company references appeared in 5,343 posts (9.2%). 23andMe was most frequently mentioned (*n* = 1,835; 3.2%), followed by AncestryDNA (*n* = 1,804; 3.1%), MyHeritage (*n* = 781; 1.3%), FamilyTreeDNA (*n* = 119; 0.2%), and LivingDNA (*n* = 41; 0.1%).

### Ancestry dispute patterns

Dispute rates varied substantially across ancestry categories. Among ancestries with ≥ 20 mentions, dispute rates ranged from 4.8% to 23.5% (Table 9). European ancestries (British, Scandinavian) showed lower relative dispute rates despite high mention frequency, suggesting greater user acceptance.

### Sentiment analysis

#### VADER sentiment scores

VADER analysis yielded a mean compound score of 0.206 (SD = 0.495), indicating overall positive sentiment. Classification by VADER thresholds revealed 45.2% positive (compound > 0.05), 42.0% neutral (− 0.05 ≤ compound ≤ 0.05), and 12.8% negative (compound < − 0.05) posts.

#### TextBlob sentiment scores

TextBlob analysis produced a mean polarity of 0.088 (SD = 0.312), confirming positive sentiment. Mean subjectivity was 0.542 (SD = 0.287), indicating moderate to high subjectivity in ancestry discussions.

#### Platform differences

**Fig. 3:**
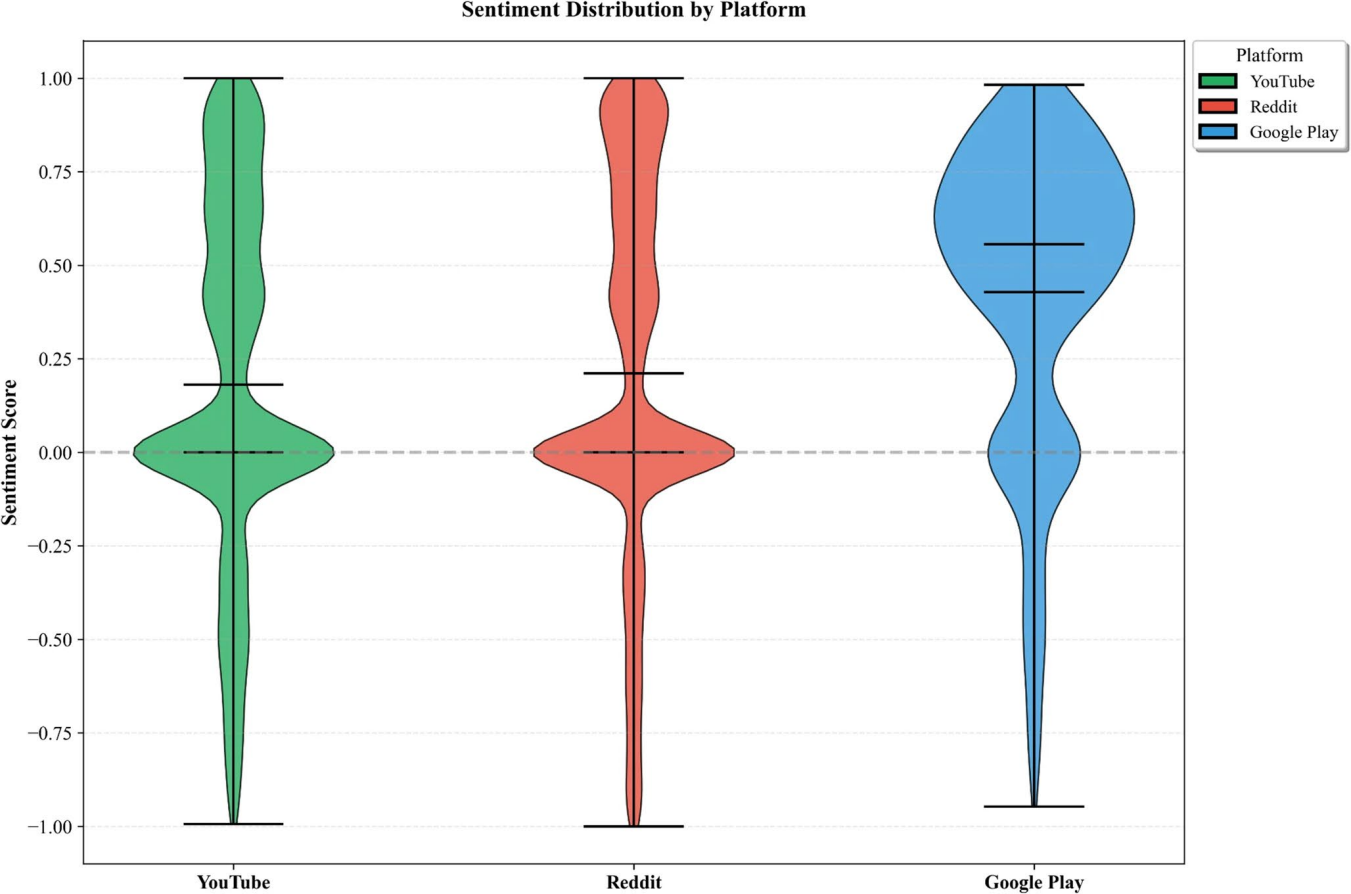
VADER compound sentiment score distribution by platform. Google Play reviews showed positive skew (M = 0.428), reflecting selection bias toward satisfied reviewers. Reddit and YouTube exhibited broader distributions with greater variance. Dashed line indicates neutral sentiment (0). Boxplots show median, interquartile range (IQR), and whiskers extending to 1.5 × IQR

Sentiment scores differed across platforms (Fig. 3). Google Play reviews exhibited the highest mean VADER score (M = 0.428, SD = 0.411), consistent with selection bias toward satisfied reviewers. Reddit posts showed moderate positive sentiment (M = 0.211, SD = 0.495). YouTube comments demonstrated slightly lower but positive sentiment (M = 0.181, SD = 0.477).

#### Sentiment by reaction type

**Fig. 4:**
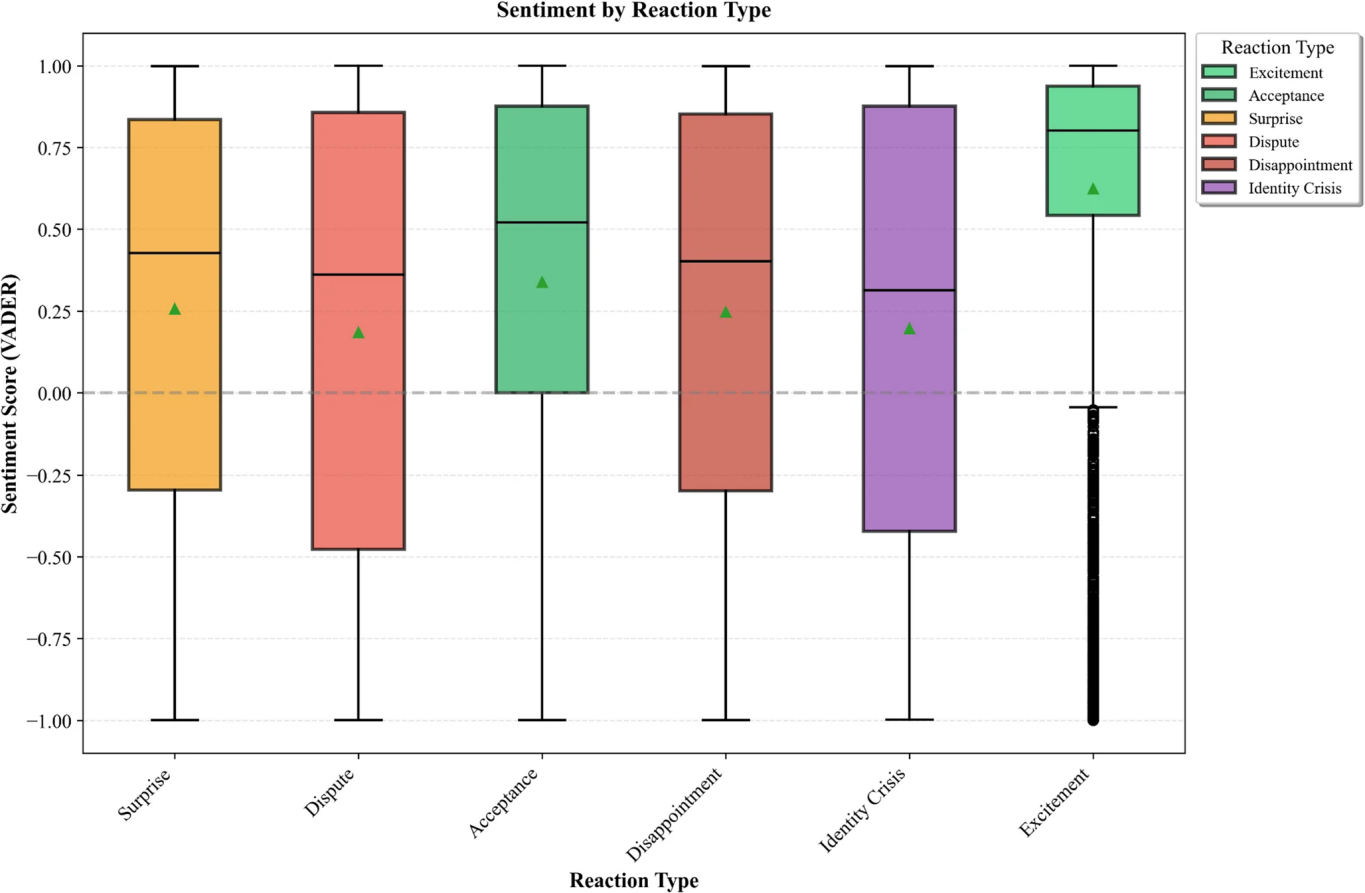
VADER compound sentiment score distribution by reaction type. Boxplots show median, interquartile range, and whiskers; green triangles indicate means; points represent outliers. Excitement showed the highest mean sentiment (M = 0.625), while dispute (M = 0.185) and identity crisis (M = 0.197) exhibited the lowest scores with wide variance, reflecting the complex emotional states associated with these reactions

Because reaction categories are inherently valence-laden, this comparison serves primarily as construct validation, assessing whether keyword-based reaction labels align with independently measured sentiment intensity rather than testing a novel association (Fig. 4; Table 7). Excitement posts showed the highest mean VADER score (M = 0.625), followed by acceptance (M = 0.339), confirming alignment between reaction labels and positive discourse. Dispute reactions were associated with lower sentiment (M = 0.185). Notably, identity crisis was the only reaction type not significantly associated with sentiment (t = 0.67, *p* = 0.505, d = 0.02; see Sect. 3.12), suggesting that identity disruption involves emotionally complex states not reducible to simple positive or negative valence.

**Table 7:** VADER sentiment scores by reaction type

Reaction	Mean VADER
Excitement	0.625
Acceptance	0.339
Surprise	0.257
Disappointment	0.248
Identity crisis	0.197
Dispute	0.185

### Topic modeling

LDA identified eight topics (Table 8). Topic 7 related directly to DNA test results. Topic 6 focused on DNA matches and family connections. Topic 1 centered on family tree research. Topic 3 related to genealogical records. The topic distribution indicates that discussions centered on result interpretation, family connections, and genealogical research.

**Table 8:** LDA topic summary

Topic	Label	Top Terms
0	General discussion	help, question, post, comment, thanks
1	Family tree research	tree, family, ancestry, data, record
2	App experience	app, test, kit, sample, waiting
3	Genealogical records	census, married, county, death, marriage
4	Platform content	video, like, people, think, know
5	Family history	grandfather, grandmother, grandparents, ancestors
6	DNA matches	dna, matches, cousin, father, mother
7	Test results	results, ancestry, dna, american, african, european

#### Sentiment by topic

To provide sentiment contrasts independent of valence-laden reaction labels, we examined mean VADER compound scores across LDA topics. Sentiment differed significantly across topics (Kruskal–Wallis H = 1152.04, *p* < 0.001, η² = 0.020). Family history narratives (Topic 5, M = 0.317, SD = 0.533) and family tree research (Topic 1, M = 0.309, SD = 0.519) showed the highest sentiment, whereas genealogical record discussions (Topic 3, M = 0.077, SD = 0.633) showed the lowest. Because topics are defined by content rather than affect, these differences reflect variation in emotional tone across discussion contexts rather than definitional overlap with reaction categories.

### Ancestry dispute rates

Among ancestries with ≥ 20 mentions, dispute rates ranged from 4.8% to 23.5% (Table 9). Turkish ancestry exhibited the highest dispute rate (23.5%; 20/85), followed by Greek (19.7%; 15/76) and Scandinavian (18.5%; 66/356). British ancestry, despite the highest mention frequency (*n* = 621), showed a relatively low dispute rate (13.8%), indicating that mention frequency does not predict dispute likelihood.

**Table 9:** Dispute rates by ancestry (≥20 mentions)

Ancestry	Total	Disputed	Rate (%)
Turkish	85	20	23.5
Greek	76	15	19.7
Scandinavian	356	66	18.5
Middle Eastern	149	27	18.1
Irish	298	52	17.4
Italian	300	49	16.3
German	219	33	15.1
British	621	86	13.8
Indian	132	14	10.6
Chinese	107	8	7.5

### Self-reported versus genetic ancestry concordance

Among 170 posts containing both self-reported ethnicity and genetic ancestry results (0.3% of corpus), concordance — defined as at least one shared normalized ancestry category — was observed in 105 cases (61.8%). In 65 cases (38.2%), self-identified ethnicity did not overlap with any genetic ancestry category. This lenient, binary definition of concordance means that even partial overlap (e.g., one shared category out of several) counted as a match; stricter definitions requiring proportional overlap would likely yield lower concordance rates.

### Inferential statistics

#### Association between ancestry results and reactions

Chi-square tests revealed significant associations between the presence of extractable ancestry results and emotional reactions. Posts containing ancestry percentages were significantly more likely to express surprise (χ² = 520.67, df = 1, *p* < 0.001, Cramér’s V = 0.095) and dispute (χ² = 201.63, df = 1, *p* < 0.001, Cramér’s V = 0.059). Source platform was significantly associated with dispute expression (χ² = 429.31, df = 2, *p* < 0.001, Cramér’s V = 0.086). All effect sizes were small.

#### Platform differences in reactions

Chi-square tests confirmed significant platform differences across all reaction types (Table 10). Acceptance showed the largest platform effect (χ² = 1538.24, Cramér’s V = 0.163), followed by excitement (χ² = 503.87, Cramér’s V = 0.093).

**Table 10:** Chi-square tests for platform × reaction associations

Reaction	χ²	df	*p*	Cramér’s V
Acceptance	1538.24	2	< 0.001	0.163
Excitement	503.87	2	< 0.001	0.093
Dispute	429.31	2	< 0.001	0.086
Disappointment	296.75	2	< 0.001	0.071
Surprise	53.74	2	< 0.001	0.030
Identity crisis	51.76	2	< 0.001	0.030

#### Platform sentiment comparison

One-way ANOVA revealed significant differences in VADER compound scores across platforms (F = 128.94, *p* < 0.001, η² = 0.004). Despite statistical significance, the small effect size indicates minimal practical difference. Tukey HSD post-hoc tests confirmed all pairwise differences were significant (*p* < 0.001).

#### Sentiment by reaction type

Because reaction categories are affect-labeled constructs, these comparisons are interpreted as construct validation rather than independent hypothesis tests. Independent samples t-tests compared sentiment scores between posts with and without each reaction type (Table 11). Excitement showed the largest effect (t = − 69.01, *p* < 0.001, Cohen’s d = 0.98), confirming that this keyword-based label aligns with independently measured positive sentiment. Identity crisis was the only reaction not significantly associated with sentiment (t = 0.67, *p* = 0.505, d = 0.02), indicating that identity disruption reflects emotionally complex discourse not captured by unidimensional sentiment polarity.

**Table 11:** T-tests comparing sentiment scores by reaction presence

Reaction	With M (SD)	Without M (SD)	t	*p*	d
Excitement	0.625 (0.472)	0.163 (0.470)	−69.01	< 0.001	0.98
Acceptance	0.339 (0.598)	0.192 (0.474)	−21.30	< 0.001	0.30
Surprise	0.257 (0.628)	0.203 (0.480)	5.91	< 0.001	0.11
Disappointment	0.248 (0.637)	0.204 (0.483)	−3.96	< 0.001	0.09
Dispute	0.185 (0.684)	0.208 (0.467)	3.14	0.002	0.05
Identity crisis	0.197 (0.685)	0.206 (0.484)	0.67	0.505	0.02

#### Non-parametric sensitivity analyses

Kruskal-Wallis H test comparing sentiment across all reaction types was highly significant (H = 1430.53, *p* < 0.001, η² = 0.063), indicating that reaction type explains approximately 6.3% of variance in sentiment scores.

#### Correlation analysis

**Fig. 5:**
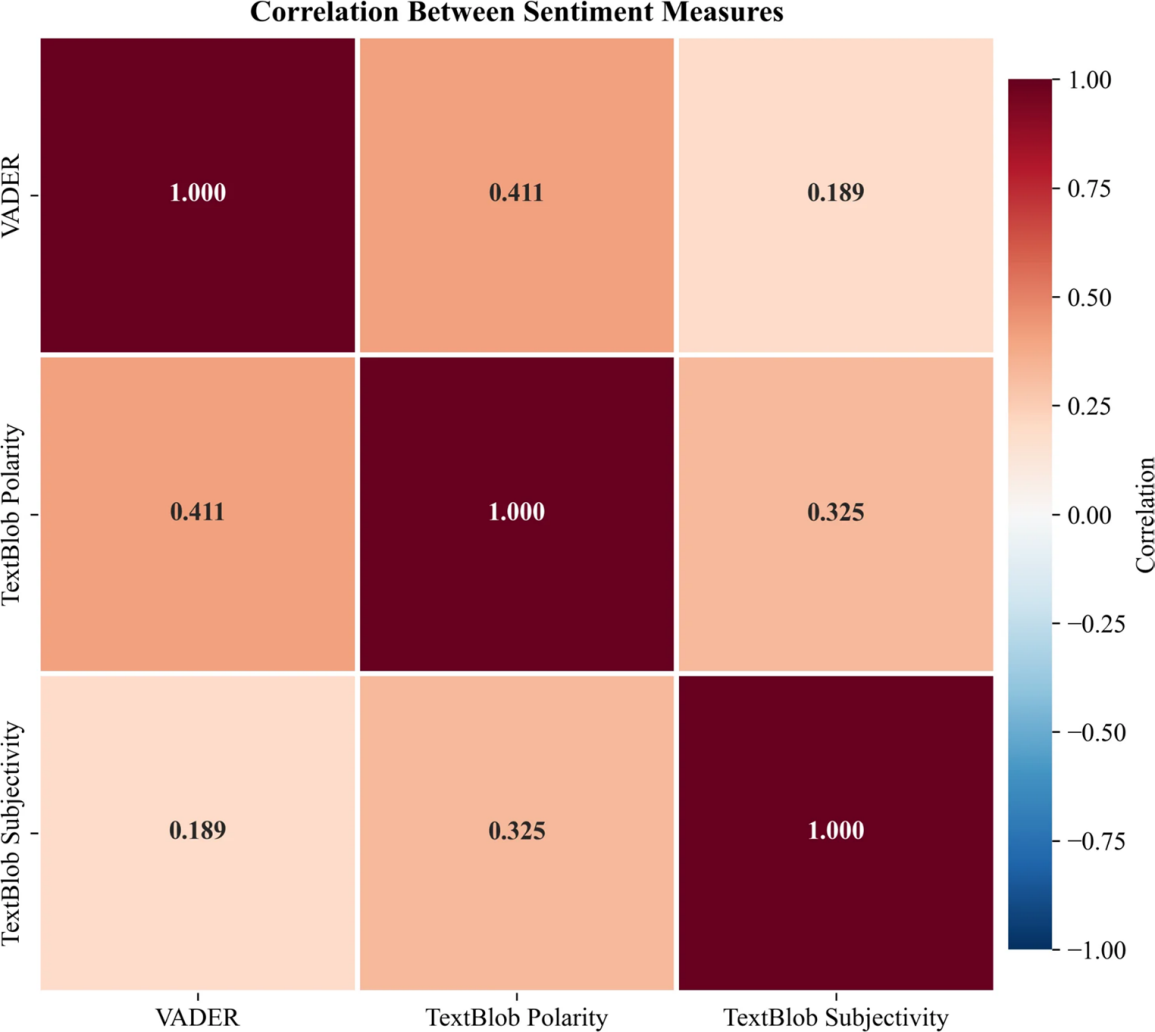
Correlation matrix of sentiment measures. VADER compound and TextBlob polarity showed moderate convergent validity (*r* = 0.411), indicating these methods capture related but distinct aspects of sentiment and supporting their complementary use. TextBlob subjectivity correlated weakly with VADER compound (*r* = 0.189) and moderately with TextBlob polarity (*r* = 0.325), confirming that subjectivity represents a separate construct from sentiment valence. Diagonal shows variable distributions; lower triangle shows scatter plots; upper triangle shows Pearson correlation coefficients

Consistent with the methodological triangulation described in Sect. 2.3, VADER compound and TextBlob polarity were moderately correlated (*r* = 0.411, *p* < 0.001), confirming convergent validity between the two sentiment measures (Fig. 5). VADER compound and TextBlob subjectivity showed a weaker association (*r* = 0.189), while TextBlob polarity and subjectivity were moderately correlated (*r* = 0.325), indicating that subjectivity captures a related but distinct dimension of discourse.

#### Predictive modeling

**Fig. 6:**
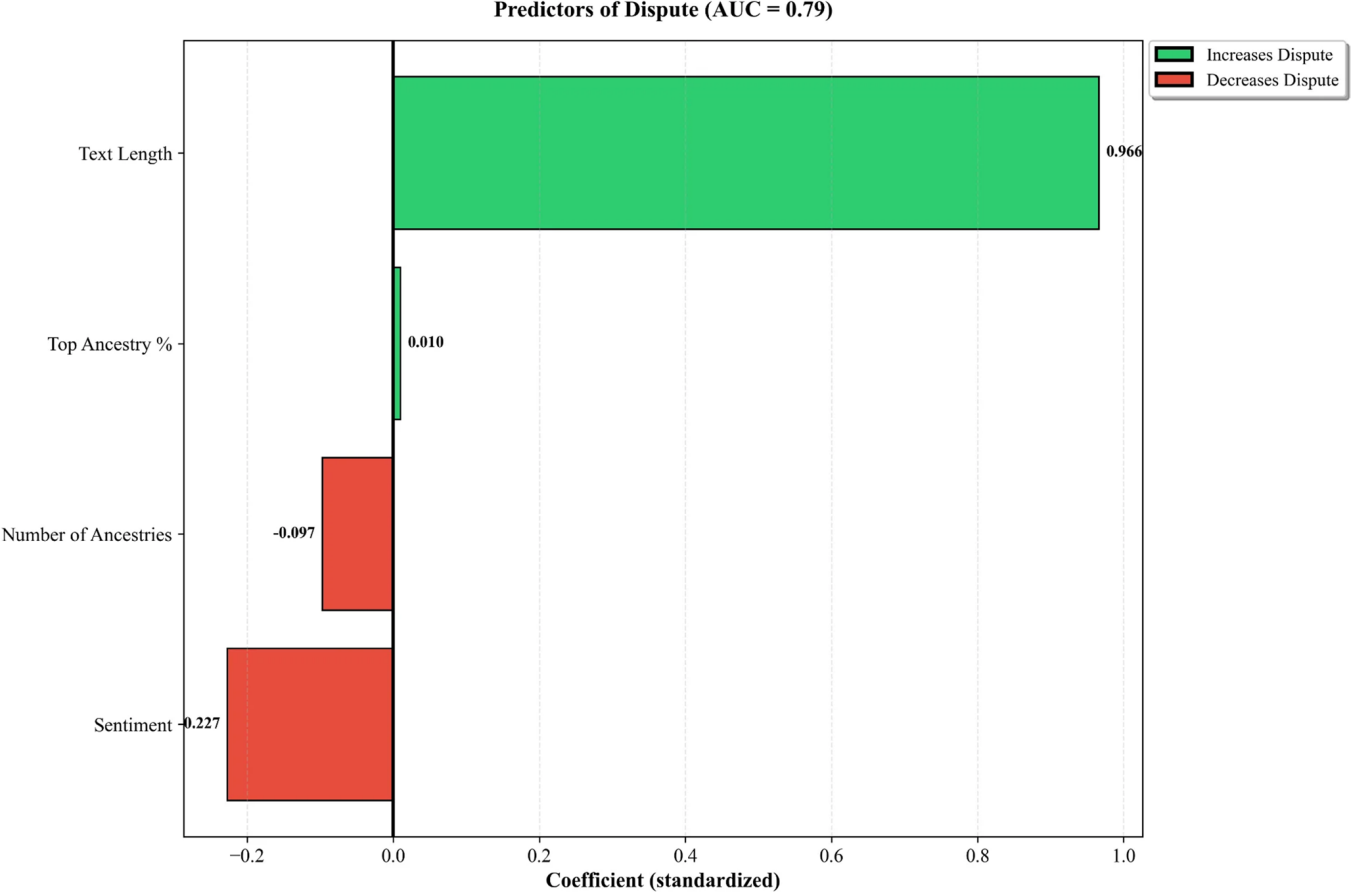
Standardized logistic regression coefficients predicting dispute expression (AUC = 0.789, 5-fold cross-validation). Text length was the dominant predictor (β = 0.966), indicating that longer posts were more likely to contain dispute. Negative VADER compound sentiment predicted dispute (β = −0.227), while number of ancestries mentioned showed a small negative effect (β = −0.097). Top ancestry percentage had minimal predictive value (β = 0.010). Error bars represent 95% confidence intervals

Logistic regression predicting dispute achieved good discriminative performance (AUC = 0.789, SD = 0.029, 5-fold CV; *n* = 4,379) (Fig. 6). Text length was the strongest predictor (β = 0.966), indicating that longer posts were more likely to contain dispute. VADER compound sentiment was negatively associated with dispute (β = −0.227). Number of ancestries mentioned showed a small negative effect (β = −0.097), while top ancestry percentage had minimal predictive value (β = 0.010).

### Classifier validation

Manual validation on 299 annotated posts demonstrated strong performance of the keyword-based reaction classifier (Table 12). Macro-averaged F1 was 0.897 (precision = 0.829, recall = 0.978), with a mean Cohen’s κ of 0.871, indicating near-perfect agreement between the keyword classifier and human judgment (Landis and Koch 1977). Surprise achieved the highest performance (F1 = 0.953, κ = 0.943), while dispute (F1 = 0.859, κ = 0.819) and excitement (F1 = 0.862, κ = 0.829) showed the lowest precision due to false positives where reaction-associated keywords appeared in non-reaction contexts. Recall was uniformly high across all categories (≥ 0.962), indicating that the classifier captures the vast majority of true reactions. Disappointment achieved perfect recall (1.000) with no false negatives. Across 1,794 reaction–post evaluations, there were 313 true positives, 66 false positives, 7 false negatives, and 1,408 true negatives, yielding an overall accuracy of 95.9%.

**Table 12:** Manual validation of keyword-based reaction classifier (*n* = 299)

Reaction	TP		FP		FN	TN	Precision	Recall	F1	Cohen’s κ
Surprise	51		3		2	243	0.944	0.962	0.953	0.943
Dispute	58		17		2	222	0.773	0.967	0.859	0.819
Acceptance	70		13		1	215	0.843	0.986	0.909	0.878
Disappointment	46		10		0	243	0.821	1.000	0.902	0.882
Identity Crisis	38		8		1	252	0.826	0.974	0.894	0.877
Excitement	50		15		1	233	0.769	0.980	0.862	0.829
**Micro-average**	**313**		**66**		**7**	**1408**	**0.826**	**0.978**	**0.896**	**0.871**
**Macro-average**	—		—		—	—	**0.829**	**0.978**	**0.897**	**0.871**

*TP* true positive, *FP* false positive, *FN* false negative, *TN* true negative. κ ≥ 0.81 = near-perfect agreement (Landis and Koch 1977)

## Discussion

This study provides a multi-platform computational analysis of consumer reactions to genetic ancestry testing. Analyzing 58,133 user-generated posts from Reddit, YouTube, and Google Play, we developed a six-category reaction taxonomy, quantified ancestry-specific dispute patterns, and built a predictive model for dispute expression. Our findings reveal systematic variation in how consumers discuss ancestry results across platforms and ancestry categories, with implications for understanding the social construction of genetic identity.

### Reaction patterns and platform differences

Acceptance (9.5%) and excitement (9.4%) were the most prevalent reactions in our corpus, followed by dispute (8.6%), surprise (5.3%), disappointment (3.6%), and identity crisis (2.3%). This pattern aligns with prior survey research documenting generally positive consumer experiences with DTC ancestry testing. Rubanovich et al. (2021) reported that fewer than 1% of users experienced distress from ancestry results, consistent with our finding that identity crisis—the most severe reaction category—occurred in only 2.3% of posts. Similarly, Stewart et al. (2018) concluded that DTC testing produces low levels of psychological distress at the population level, a pattern reflected in the predominance of positive reactions in our data.

Platform differences emerged as a significant finding. Reddit exhibited the highest dispute rates (10.2%) and acceptance rates (12.8%), while Google Play showed elevated excitement (29.6%). These patterns likely reflect platform-specific selection biases and affordances. Reddit’s pseudonymous discussion forums may encourage candid expression of skepticism and detailed engagement with results, while app store reviews attract users motivated to share strong positive or negative experiences. YouTube comments, attached to “DNA reveal” videos emphasizing dramatic reactions, showed intermediate patterns. These findings extend prior single-platform analyses (Yin et al. 2020; Toussaint et al. 2022) by demonstrating that platform context systematically shapes how consumers discuss genetic ancestry.

### Ancestry-specific dispute patterns

Dispute rates varied substantially across ancestry categories, ranging from 7.5% (Chinese) to 23.5% (Turkish). This finding represents a contribution to the computational literature, which has not previously quantified ancestry-specific dispute patterns. Turkish (23.5%), Greek (19.7%), and Scandinavian (18.5%) ancestries showed the highest dispute rates, while Chinese (7.5%) and Indian (10.6%) ancestries were least frequently contested. Notably, British ancestry—despite being the most frequently mentioned (*n* = 621)—showed a relatively low dispute rate (13.8%), indicating that mention frequency does not predict dispute likelihood.

These patterns may reflect several factors. First, regional ambiguity in genetic reference panels may contribute to higher dispute rates for geographically proximate populations (e.g., Turkish, Greek, Middle Eastern). Users may question results that blur distinctions important to their identity. Second, family narratives that emphasize specific ancestries may conflict with genetic estimates that distribute ancestry across multiple categories. Third, cultural and political dimensions of ethnic identity—particularly in regions with contested histories—may heighten skepticism toward genetic categorization. Walajahi et al. (2019) documented similar concerns regarding Native American ancestry claims, noting that DTC results can conflict with tribal enrollment criteria and cultural definitions of belonging. Our quantitative findings complement this qualitative work by demonstrating that ancestry-specific dispute patterns are measurable at scale.

### Sentiment and reaction types

Overall sentiment was positive (M = 0.206), consistent with Toussaint et al.‘s (2022) finding of “neutral-to-positive attitudes” in YouTube comments. Sentiment comparisons across reaction types served primarily as construct validation, confirming that keyword-based labels align with independently measured affective intensity. The large effect size for excitement (Cohen’s d = 0.98) confirms that this category captures genuinely positive discourse. The non-significant association between identity crisis and sentiment (*p* = 0.505, d = 0.02) represents a substantively meaningful exception, indicating that identity disruption involves emotionally complex states not reducible to simple valence.

Sentiment differences across LDA topics provided more independent explanatory contrasts. Narrative-oriented topics exhibited higher mean sentiment, whereas record-focused discussions were more neutral in tone. Although effect sizes were modest (η² = 0.020), this pattern indicates that emotional tone varies systematically across content-defined discussion contexts rather than being solely determined by reaction labels.

The moderate correlation between VADER and TextBlob polarity (*r* = 0.411) demonstrates convergent validity while suggesting these methods capture related but distinct aspects of sentiment. This finding supports methodological triangulation in computational text analysis, as different sentiment tools may emphasize different linguistic features. The weak correlation between sentiment valence and subjectivity (*r* = 0.189) confirms that subjectivity represents a separate construct from polarity. The high mean subjectivity across the corpus (M = 0.542) indicates that ancestry discussions are predominantly framed as personal, experiential narratives rather than factual or informational exchanges. This distinction is substantively relevant for understanding specific reaction types. Identity crisis posts, which showed no significant association with sentiment polarity, may nonetheless reflect highly subjective discourse in which users narrate personal experiences of disruption without expressing clearly positive or negative evaluations. Dispute posts may similarly combine negative polarity with high subjectivity, reflecting personally invested skepticism rather than detached factual criticism. Subjectivity thus provides an interpretive dimension that polarity alone cannot capture, distinguishing between personal identity negotiation and impersonal informational discourse within ancestry discussions.

### Self-reported versus genetic ancestry concordance

Among posts containing both self-reported ethnicity and genetic ancestry results, concordance was 61.8%, using a lenient definition that counted any overlapping normalized ancestry category as a match. The 38.2% discordance rate should be interpreted with caution given the small sample size (*n* = 170) and the nature of the measure. This figure reflects divergence between categorical labels extracted through automated text processing rather than direct evidence of experiential identity conflict or negotiation. Nonetheless, the observed discordance is consistent with theoretical perspectives emphasizing the socially constructed nature of ethnic identity (Morning 2011) and with qualitative findings that consumers selectively incorporate genetic results into pre-existing identity narratives (Roth and Ivemark 2018). Roth and Ivemark (2018) found that white respondents often embraced unexpected ancestries while nonwhite respondents maintained existing identifications; our concordance analysis provides preliminary, descriptive evidence that genetic results frequently diverge from self-identification in online discourse, though the extent to which this divergence reflects active identity negotiation cannot be determined from text data alone.

### Predictors of dispute expression

Our logistic regression model achieved good discriminative performance (AUC = 0.79), identifying text length as the dominant predictor of dispute expression (β = 0.966). Text length is a structurally powerful predictor in most text classification tasks, and its dominance here likely reflects in part an opportunity effect: longer posts contain more words and thus more chances to include dispute keywords. However, the strength of the association also suggests a substantive component, as users questioning their results may tend to write longer posts explaining their skepticism, providing family history, or detailing discrepancies across testing companies. These explanations are not mutually exclusive, and the present model cannot distinguish between them. Negative sentiment also predicted dispute (β = −0.227), confirming the intuitive association between skepticism and negative affect. The small negative effect of number of ancestries mentioned (β = −0.097) may indicate that users with more complex results focus on interpretation rather than dispute, or that dispute tends to center on specific contested ancestries rather than overall result complexity.

This model is intended primarily for prediction rather than for causal explanation of why users dispute their results. The dominance of text length and the limited feature set constrain the model’s utility for understanding underlying motivations for dispute. Nonetheless, the model demonstrates that dispute expression has identifiable textual signatures amenable to computational detection. Any practical application, such as flagging posts for genetic counseling support or monitoring platform discourse, would require validation with richer feature sets and human review.

### Theoretical implications

Our findings contribute to theoretical understanding of genetic ancestry as a socially negotiated phenomenon. The substantial variation in dispute rates across ancestry categories demonstrates that genetic results are not passively received but actively evaluated against cultural expectations, family narratives, and identity commitments. This supports constructivist perspectives on genetic identity (Roth and Ivemark 2018; Lang and Winkler 2021) while providing quantitative evidence that the intensity of identity negotiation varies systematically by ancestry category.

Platform differences in reaction patterns highlight the role of communicative context in shaping how genetic information is discussed. The distinct reaction profiles across Reddit, YouTube, and Google Play suggest that platform affordances—anonymity, audience, content format—influence not only whether users share ancestry experiences but how they frame those experiences emotionally and cognitively. This finding extends media studies perspectives to the domain of consumer genomics, suggesting that understanding public engagement with genetic information requires attention to the platforms mediating that engagement.

### Practical implications

Our findings have several practical implications. For DTC genetic testing companies, the ancestry-specific dispute patterns suggest that certain results may benefit from enhanced explanation or contextualization. Ancestries with high dispute rates (Turkish, Greek, Middle Eastern) might warrant additional information about reference panel composition, regional genetic overlap, or the distinction between genetic ancestry and ethnic identity. Companies might also monitor platform-specific discourse to understand how their results are received across different user communities.

For genetic counselors, our reaction taxonomy provides a framework for anticipating consumer responses. The predominance of acceptance and excitement suggests that most users have positive experiences, but the meaningful prevalence of dispute (8.6%) and identity crisis (2.3%) indicates that some users require support in interpreting results that conflict with expectations. The 38.2% discordance between self-reported and genetic ancestry highlights the frequency with which users may need guidance in reconciling genetic information with existing identity narratives.

For researchers studying public engagement with genomics, our methodology demonstrates the feasibility of large-scale computational analysis of consumer discourse. The combination of reaction taxonomy, ancestry extraction, sentiment analysis, and predictive modeling provides a template for examining how genetic information is discussed in online contexts, with potential applications to health-related genetic testing, pharmacogenomics, and other domains.

### Limitations

Several limitations warrant consideration. First, our reaction classification relied on keyword-based pattern matching, which may miss reactions expressed through indirect language, sarcasm, or context-dependent phrasing. Manual validation on a stratified subsample of 299 posts yielded a macro-averaged F1 of 0.897 and mean Cohen’s κ of 0.871, indicating that the keyword classifier achieves near-perfect agreement with human judgment. However, precision was lower than recall (0.829 vs. 0.978), indicating a tendency toward false positives, particularly for dispute and excitement where reaction-associated keywords sometimes appeared in non-reaction contexts. This suggests that corpus-level prevalence estimates for these categories may be slightly inflated. Validation was performed by a single annotator; inter-annotator reliability with a second independent coder would further strengthen confidence in the classification scheme. Machine learning classifiers trained on annotated data might improve classification accuracy, though they would require substantial annotation effort. Second, our analysis was limited to English-language content, excluding non-English discussions that may reflect different cultural relationships to genetic ancestry. Third, the cross-sectional design precludes analysis of how reactions evolve over time as users integrate results into their identities or receive updated estimates from companies.

Fourth, platform-specific sampling strategies may introduce selection biases. Reddit posts were drawn from ancestry-focused subreddits where engaged users congregate, YouTube comments were attached to videos selected by search queries, and Google Play reviews reflect users motivated to provide feedback. These samples may not represent the broader population of DTC ancestry testing consumers. Fifth, we could not verify the accuracy of self-reported genetic results; users may misremember, misreport, or selectively share their results. Sixth, the ancestry extraction patterns captured only a subset of posts with extractable percentages (7.5%), limiting generalizability of ancestry-specific findings. Seventh, the reaction categories used in this study represent operational discourse labels derived from keyword patterns rather than validated measures of psychological states. As a consequence, some sentiment-reaction analyses are confirmatory by design, as affect-laden labels (e.g., excitement, disappointment) are expected to align with sentiment polarity. While we have framed these comparisons as construct validation throughout the manuscript, this inherent overlap should be considered when interpreting the strength of sentiment-reaction associations. Eighth, the dominance of text length in the predictive model (β = 0.966) suggests that the model’s discriminative performance may reflect structural properties of posts, such as the increased opportunity for longer texts to contain dispute keywords, rather than underlying motivations for disputing ancestry results. This limits the explanatory value of the model and suggests that future work incorporating richer linguistic and demographic features would be needed to identify substantive predictors of dispute.

### Future directions

Future research could extend this work in several directions. Longitudinal analysis of user posting histories might reveal how reactions evolve as users engage with ancestry communities over time. Multilingual analysis could examine cultural variation in ancestry interpretation across different national and linguistic contexts. Deep learning approaches to reaction classification might capture more nuanced expressions of skepticism or identity disruption. Integration of demographic data, where available, could examine how user characteristics moderate reactions to different ancestry results. Finally, comparative analysis across different types of DTC genetic testing—ancestry versus health versus traits—might reveal domain-specific patterns in consumer response.

## Conclusion

This study provides a multi-platform computational analysis of consumer reactions to DTC genetic ancestry testing. Analyzing 58,133 posts from Reddit, YouTube, and Google Play, we developed a six-category reaction taxonomy, quantified ancestry-specific dispute patterns, and built a predictive model for dispute expression. Our findings reveal that while most consumers express positive reactions (acceptance, excitement), dispute occurs in 8.6% of posts and varies substantially by ancestry category—with Turkish (23.5%), Greek (19.7%), and Scandinavian (18.5%) ancestries most frequently contested. Platform differences in reaction patterns highlight how communicative context shapes engagement with genetic information. The 38.2% discordance between self-reported and genetic ancestry quantifies the frequency with which genetic results diverge from social identity, while our predictive model (AUC = 0.79) identifies text length and negative sentiment as key predictors of dispute.

These findings illustrate how ancestry results are discussed across different online platforms. As genetic ancestry testing continues to grow, computational methods offer valuable tools for understanding public discourse at scale, complementing survey and interview approaches with breadth and ecological validity.

## Data Availability

Raw text data cannot be redistributed due to the terms of service of the source platforms (Reddit, YouTube, Google Play). Post identifiers enabling independent data re-collection, all analysis code, keyword lexicons for reaction classification, ethnicity normalization mappings, and regular expression patterns are available at [https://github.com/sarabehnamian/ancestry_nlp]. Aggregated results are available from the corresponding author on reasonable request.
